# Impact of abdominal obesity on outcomes of catheter ablation in Korean patients with atrial fibrillation

**DOI:** 10.1111/ijcp.14696

**Published:** 2021-08-06

**Authors:** Wern Yew Ding, Pil‐Sung Yang, Eunsun Jang, Dhiraj Gupta, Jung‐Hoon Sung, Boyoung Joung, Gregory Y. H. Lip

**Affiliations:** ^1^ Liverpool Centre for Cardiovascular Science University of Liverpool and Liverpool Heart & Chest Hospital Liverpool UK; ^2^ Department of Cardiology CHA Bundang Medical Center CHA University Seongnam Republic of Korea; ^3^ Department of Cardiology Severance Cardiovascular Hospital Yonsei University College of Medicine Seoul Republic of Korea; ^4^ Aalborg Thrombosis Research Unit Department of Clinical Medicine Aalborg University Aalborg Denmark

## Abstract

**Background:**

Effects of abdominal obesity on outcomes of atrial fibrillation (AF) ablation remains ill‐defined. Here, we evaluated the impact of abdominal obesity on the long‐term efficacy and safety of catheter AF ablation among Korean patients.

**Methods:**

We utilised the Korean National Health Insurance Service database to identify patients who underwent AF ablation. Abdominal obesity was defined as waist circumference ≥90 cm (males) and ≥85 cm (females). The primary endpoint was AF recurrence and secondary endpoints were ischaemic stroke, intracranial haemorrhage and death. Additionally, safety endpoints of peri‐procedural complications were studied.

**Results:**

Among 5397 patients (median age 58 [IQR 51‐65] years; 23.6% females), abdominal obesity was present in 1759 (32.6%). The rate of AF recurrence was not statistically different between the groups at 1‐year (10.3 vs 8.7 events/100‐PYs, *P* = .078), though abdominal obesity was associated with significantly higher rates of AF recurrence at 3‐year (7.6 vs 6.3 events/100‐PYs, *P* = .008) and 6‐year (6.3 vs 5.2 events/100‐PYs, *P* = .004) follow‐ups. Kaplan‐Meier survival analysis found significantly higher rates of AF recurrence in patients with obesity based on body mass index (BMI) and waist circumference (log‐rank for trend *P* = .006). Using multivariable regression analysis, obesity by both BMI and waist circumference was an independent predictor for AF recurrence (HR 1.21 [95% CI, 1.05‐1.40]), after accounting for other risk factors.

There was a trend for increased rates of ischaemic stroke at 3‐year and 6‐year follow‐ups in patients with abdominal obesity. Furthermore, this group of patients had a greater rate of intracranial haemorrhage. All‐cause death was comparable between both groups. Total peri‐procedural complications were not associated with abdominal obesity.

**Conclusion:**

Abdominal obesity as indicated by waist circumference was associated with a greater burden of concomitant diseases and an independent risk factor for long‐term redo AF intervention following catheter ablation but had no effects on total peri‐procedural complications.

**What’s known**

- Obesity is strongly linked to atrial fibrillation through various mechanisms.
- The best anthropometric surrogate of visceral adiposity is abdominal obesity, as defined by waist circumference, which has been shown to be a better predictor than body mass index for cardiovascular risk factors, disease and mortality.
- Nonetheless, there is limited evidence on the influence of abdominal obesity on outcomes of catheter ablation for atrial fibrillation.

**What’s new**

- Based on our study, we found that abdominal obesity (defined as waist circumference ≥90 cm for males and ≥85 cm for females) was an independent risk factor for long‐term recurrence of atrial fibrillation following catheter ablation.
- Furthermore, abdominal obesity was associated with significantly greater risk of long‐term recurrence of atrial fibrillation after index ablation even among obese patients, as defined using body mass index.
- Interestingly, the presence of abdominal obesity was not related to an excess of peri‐procedural complications.

## 1 INTRODUCTION

Atrial fibrillation (AF) is a significant public health problem that is associated with a greater risk of stroke and heart failure, reduced quality of life and increased mortality. The increasing prevalence of the condition may, in part, be attributable to rising trends of obesity. A population‐based study demonstrated a global increase in mean body mass index (BMI) over the past four decades in both males (21.7‐24.2 kg/m^2^) and females (22.1‐24.4 kg/m^2^).^1^ Furthermore, the authors reported that the age‐standardised prevalence of obesity increased significantly over the same duration (males: 3.2%‐10.8%; females: 6.4%‐14.9%).

Obesity is characterised by an accumulation of adipose tissue, distributed into two main compartments (subcutaneous adipose tissue and visceral adipose tissue) with different metabolic characteristics. There has been more attention on visceral adipose tissue due to its strong association with various cardiovascular pathologies such as AF. The role of obesity in AF per se is complex and likely multifactorial. For example, obesity has been linked to increased left atrial size and decreased left ventricular diastolic function,^2^ leading to higher left atrial pressure. A separate mechanism by which obesity may be linked to AF is through the development of obstructive sleep apnoea. Overall, the aforementioned factors are known to promote the initiation and maintenance of AF.

The best anthropometric surrogate of visceral adiposity is abdominal obesity, as defined by waist circumference. This is supported by the fact that waist circumference has been shown to be a better predictor than BMI for cardiovascular risk factors, disease and mortality.^3^ Furthermore, waist circumference is an independent component of metabolic syndrome and has been associated with an increased risk of AF.^4^ In a meta‐analysis of 29 prospective studies, Aune et al^5^ found that every 10 cm increase in waist circumference was associated with an 18% greater relative risk of incident AF.

Nonetheless, there is limited evidence on the influence of abdominal obesity on AF ablation, especially in relation to long‐term arrhythmia recurrence. In this study, we evaluate the impact of abdominal obesity on the long‐term efficacy and safety outcomes of catheter ablation for AF.

## 2 METHODS

This study was a retrospective cohort analysis using the national health claims established by the National Health Insurance Service (NHIS) of Korea. The national health insurance system in South Korea was established in 1963 and requires compulsory participation from its citizens. At present, the NHIS is responsible for managing all Korean health service databases and discharging the national health examination programs which include a general medical examination for insured employees, or self‐employed persons aged over 40 years and their dependents. These examinations are recommended at least biennially. This study was approved by the institutional review board, and the requirement for informed consent was waived.

### 2.1 Study population

From the Korean NHIS database covering a population 51.5 million inhabitants, 834 735 adult patients (≥18 years) were newly diagnosed with AF from 2006 to 2015. Atrial fibrillation was identified using the International Classification of Disease 10th revision code I48. This method has previously been validated in the NHIS database with a positive predictive value of 94.1%.^6^ Subsequently, our study population included only those who underwent catheter ablation for AF. Catheter ablation for AF was identified using the corresponding NHIS procedure codes for AF ablation (M6542 or M6547) with an admission diagnosis of AF. No specific exclusion criteria were employed.

Patients were categorised into two groups based on the presence or absence of abdominal obesity (defined as waist circumference ≥90 cm for males and ≥85 cm for females according to 2018 Guideline of Korean Society for the Study of Obesity).^7^ Body mass index subgroups were based on the World Health Organization guidelines for the Asia‐Pacific region (defined as underweight <18.5 kg/m^2^; normal 18.5‐22.9 kg/m^2^; overweight 23.0‐24.9 kg/m^2^; and obese ≥25.0 kg/m^2^).^8^

### 2.2 Covariates

Information regarding comorbid conditions was obtained from inpatient and outpatient hospital diagnoses. Baseline comorbidities were defined using medical claims and prescription medications before the index ablation. The patients were considered to have comorbidities when the condition was a discharge diagnosis or was confirmed at least twice in an outpatient setting, similar to previous studies using Korean NHIS data.

### 2.3 Clinical outcome events and assessments

The primary endpoint was AF recurrence after index ablation which was determined using surrogate markers of cardioversion or repeat AF ablation. Secondary endpoints were ischemic stroke, intracranial haemorrhage and death. These endpoints were evaluated at 1‐year, 3‐year and 6‐year follow‐ups. The definitions of clinical outcomes are presented in Table S1. Mortality data were obtained from the Korean National Statistical Office. Safety endpoints of peri‐procedural complications included pericardial effusion, cardiac tamponade, in‐hospital stroke or transient ischaemic attack, vascular complication requiring intervention, complete atrioventricular nodal block, unplanned cardiac or vascular surgery, myocardial infarction, atrio‐oesophageal fistula and phrenic nerve paralysis. The definitions of peri‐procedural complications are presented in Table S2.

### 2.4 Statistical analyses

The normality of continuous variables was assessed using Kolmogorov‐Smirnov test. Variables with a normal distribution were presented with mean and standard deviations (SDs), and tested for differences with *t* test. Variables without normal distribution were presented with median and interquartile range (IQR), and tested for differences with Mann‐Whitney U test. Categorical variables were presented with count and percentage and tested for differences with chi‐squared or Fisher's exact test.

Event rates per 100 patient‐years (PYs) were calculated for each study endpoint in the overall cohort and for subsets of patients according to heart failure status and age. Plots of Kaplan‐Meier curves for study outcomes were performed and survival distributions were compared using log‐rank test. Multivariable cox regression analyses were undertaken to identify independent predictors of AF recurrence following ablation. Multivariable cox regression models for the outcome of interest were created by including covariates that had a univariate significance of *P* < .10 for the outcome.

Plots of hazard ratios for the recurrence of AF after index ablation according to BMI and waist circumference were adjusted for age, sex, AF duration, cardiovascular implantable electronic device implantation, valvular heart disease, heart failure, hypertrophic cardiomyopathy, previous ischaemic stroke or transient ischaemic attack, previous myocardial infarction, hypertension, diabetes mellitus, chronic kidney disease, chronic obstructive pulmonary disease, liver disease, malignancy, dyslipidaemia, sleep apnoea, hypothyroidism and hyperthyroidism. Because of multicollinearity, waist circumference was excluded in the BMI model and BMI was excluded in the waist circumference model.

A two‐sided *P* value of less than .05 was considered statistically significant. Analyses were performed using SAS version 9.3 (SAS Institute) and R version 3.3.2 (The R Foundation, www.R‐project.org).

## 3 RESULTS

**FIGURE 1:**
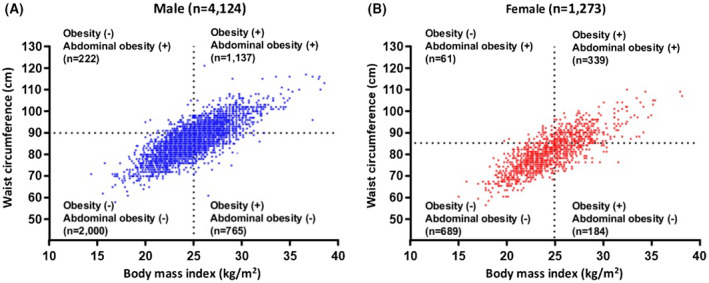
Patient distribution according to obesity (body mass index ≥25 kg/m^2^) and abdominal obesity (waist circumference ≥90 cm for males and ≥85 cm for females) in (A) males and (B) females

There was a total of 5397 patients (median age 58 [51‐65] years) who had catheter AF ablation which comprised of 1273 (23.6%) females (Table 1). Abdominal obesity was present in 1759 (32.6%) patients who had a higher median BMI compared with patients without abdominal obesity (27.0 [25.6‐28.6] kg/m^2^ vs 23.6 [22.2‐25.0] kg/m^2^, *P* < .001). Of the patients with abdominal obesity, 83.9% were categorised as obese based on BMI. The distribution of patients with obesity based on BMI and waist circumference is shown in Figure 1.

**TABLE 1:** Baseline characteristics based on abdominal obesity

	Total (n = 5397)	Abdominal obesity (−) (n = 3638)	Abdominal obesity (+) (n = 1759)	*P* value
Age (y), median (IQR)	58 (51‐65)	57 (50‐64)	58 (52‐65)	.007
Females, n (%)	1273 (23.6%)	873 (24.0%)	400 (22.7%)	.325
Waist circumference (cm), median (IQR)	85 (80‐90)	82 (78‐85)	93 (90‐96)	<.001
Body mass index (kg/m^2^), median (IQR)	24.6 (22.9‐26.5)	23.6 (22.2‐25.0)	27.0 (25.6‐28.6)	<.001
Body mass index subgroups, n (%)				
Underweight	50 (0.9%)	50 (1.4%)	0 (0.0%)	<.001
Normal weight	1343 (24.9%)	1311 (36.0%)	32 (1.8%)	<.001
Overweight	1579 (29.3%)	1328 (36.5%)	251 (14.3%)	<.001
Obese	2425 (44.9%)	949 (26.1%)	1476 (83.9%)	<.001
eGFR (mL/min/1.73 m^2^), median (IQR)	81.2 (69.2‐93.7)	81.8 (70.0‐94.4)	79.2 (68.1‐92.4)	<.001
AF duration (months), median (IQR)	27 (8‐63)	27 (8‐61)	29 (8‐65)	.367
Concomitant disease, n (%)				
Anaemia	502 (9.3%)	348 (9.6%)	154 (8.8%)	.358
Hypertrophic cardiomyopathy	100 (1.9%)	68 (1.9%)	32 (1.8%)	.984
Chronic kidney disease	196 (3.6%)	117 (3.2%)	79 (4.5%)	.023
COPD	1112 (20.6%)	691 (19.0%)	421 (23.9%)	<.001
Dementia	37 (0.7%)	21 (0.6%)	16 (0.9%)	.226
Diabetes mellitus	711 (13.2%)	428 (11.8%)	283 (16.1%)	<.001
Heart failure	1905 (35.3%)	1198 (32.9%)	707 (40.2%)	<.001
Hypertension	4572 (84.7%)	3001 (82.5%)	1571 (89.3%)	<.001
Hypothyroidism	869 (16.1%)	586 (16.1%)	283 (16.1%)	.999
Hyperthyroidism	1028 (19.0%)	699 (19.2%)	329 (18.7%)	.682
Sleep apnoea	115 (2.1%)	63 (1.7%)	52 (3.0%)	.005
Liver disease	2424 (44.9%)	1580 (43.4%)	844 (48.0%)	.002
Malignancy	1053 (19.5%)	703 (19.3%)	350 (19.9%)	.644
Peripheral vascular disease	606 (11.2%)	378 (10.4%)	228 (13.0%)	.006
Prior comorbidities, n (%)				
Ischemic stroke or TIA	1111 (20.6%)	737 (20.3%)	374 (21.3%)	.409
Myocardial infarction	518 (9.6%)	331 (9.1%)	187 (10.6%)	.081
Haemorrhagic stroke	71 (1.3%)	49 (1.3%)	22 (1.3%)	.870
CIED implantation	106 (2.0%)	86 (2.4%)	20 (1.1%)	.002
CHA_2_DS_2_‐VASc, median (IQR)	2 (1‐3)	2 (1‐3)	2 (1‐4)	<.001
Medication use, n (%)				
Antiplatelets	4466 (82.7%)	2986 (82.1%)	1480 (84.1%)	.065
Acetylsalicylic acid	4287 (79.4%)	2870 (78.9%)	1417 (80.6%)	.166
Clopidogrel	1319 (24.4%)	831 (22.8%)	488 (27.7%)	<.001
Anticoagulation	4482 (83.0%)	2967 (81.6%)	1515 (86.1%)	<.001
Vitamin K antagonist	3891 (72.1%)	2595 (71.3%)	1296 (73.7%)	.077
Any NOAC	591 (10.9%)	372 (10.2%)	219 (12.5%)	.016
Apixaban	171 (3.2%)	112 (3.1%)	59 (3.4%)	.646
Dabigatran	239 (4.4%)	150 (4.1%)	89 (5.1%)	.134
Rivaroxaban	181 (3.4%)	110 (3.0%)	71 (4.0%)	.063
Anti‐arrhythmic agents	5183 (96.0%)	3490 (95.9%)	1693 (96.2%)	.603
Class Ic	2914 (54.0%)	2013 (55.3%)	901 (51.2%)	.005
Class III	2269 (42.0%)	1477 (40.6%)	792 (45.0%)	.002
Other treatments				
ACE inhibitor/ARB	2826 (52.4%)	1764 (48.5%)	1062 (60.4%)	<.001
Beta‐blockers	3818 (70.7%)	2499 (68.7%)	1319 (75.0%)	<.001
Non‐DHP CCB	1720 (31.9%)	1146 (31.5%)	574 (32.6%)	.421
Digoxin	924 (17.1%)	598 (16.4%)	326 (18.5%)	.060
Diuretics	2121 (39.3%)	1273 (35.0%)	848 (48.2%)	<.001
Aldosterone antagonist	561 (10.4%)	324 (8.9%)	237 (13.5%)	<.001
Statins	2386 (44.2%)	1456 (40.0%)	930 (52.9%)	<.001

Abbreviations: ACE, angiotensin‐converting enzyme; AF, atrial fibrillation; ARB, angiotensin‐receptor blocker; CCB, calcium‐channel blocker; CIED, cardiovascular implantable electronic device; COPD, chronic obstructive pulmonary disease; DHP, dihydropyridine; eGFR, estimated glomerular filtration rate; IQR, interquartile range; NOAC, non‐vitamin K oral anticoagulant; SD, standard deviation; TIA, transient ischaemic attack.

Patients with abdominal obesity were older and had increased prevalence of concomitant diseases including chronic kidney disease (*P* = .023), chronic obstructive pulmonary disease (*P* < .001), diabetes mellitus (*P* < .001), heart failure (*P* < .001), hypertension (*P* < .001), sleep apnoea (*P* = .005), liver disease (*P* = .002) and peripheral vascular disease (*P* = .006).

### 3.1 Medication use

Among patients with abdominal obesity, there was increased use of anticoagulation (*P* < .001), angiotensin‐converting enzyme inhibitor or angiotensin‐receptor blocker (*P* < .001), beta‐blockers (*P* < .001), diuretics (*P* < .001), aldosterone antagonist (*P* < .001) and statins (*P* < .001). The use of antiplatelets and antiarrhythmic agents were comparable between both groups.

### 3.2 Outcomes of AF ablation

The rate of AF recurrence following index ablation was not statistically different among patients with abdominal obesity compared with patients without abdominal obesity at 1‐year follow‐up (10.3 vs 8.7 events/100 PYs, *P* = .078), though significantly higher rates of AF recurrence were observed in the former group at 3‐year (7.6 vs 6.3 events/100 PYs, *P* = .008) and 6‐year (6.3 vs 5.2 events/100 PYs, *P* = .004) follow‐ups (Table 2).

**TABLE 2:** Effects of abdominal obesity on outcomes of atrial fibrillation ablation at 1, 3 and 6 years follow‐up

	Abdominal obesity (−) (n = 3638)		Abdominal obesity (+) (n = 1759)		*P* value
	Number of events	Event rate (% or per 100 PYs)	Number of events	Event rate (% or per 100 PYs)	
Early after AF ablation					
All‐cause early mortality^a^	8	0.2	2	0.1	.515
1‐year follow‐up					
AF recurrence	304	8.7	172	10.3	.078
Major adverse events	54	1.5	39	2.3	.051
Ischemic stroke	43	1.2	29	1.7	.161
Intracranial haemorrhage	3	0.1	10	0.6	.001
All‐cause death	19	0.5	7	0.4	.538
3‐year follow‐up					
AF recurrence	546	6.3	308	7.6	.008
Major adverse events	104	1.1	66	1.5	.062
Ischemic stroke	69	0.7	47	1.1	.055
Intracranial haemorrhage	13	0.1	13	0.3	.052
All‐cause death	40	0.4	15	0.3	.426
6‐year follow‐up					
AF recurrence	639	5.2	355	6.3	.004
Major adverse events	145	1.1	92	1.4	.021
Ischemic stroke	91	0.7	59	0.9	.052
Intracranial haemorrhage	19	0.1	19	0.3	.017
All‐cause death	62	0.4	25	0.4	.610

Abbreviations: AF, atrial fibrillation; PYs, patient‐years.

^a^ All‐cause early mortality was defined as mortality occurring either at index AF ablation admission or within 30 days after ablation.

**FIGURE 2:**
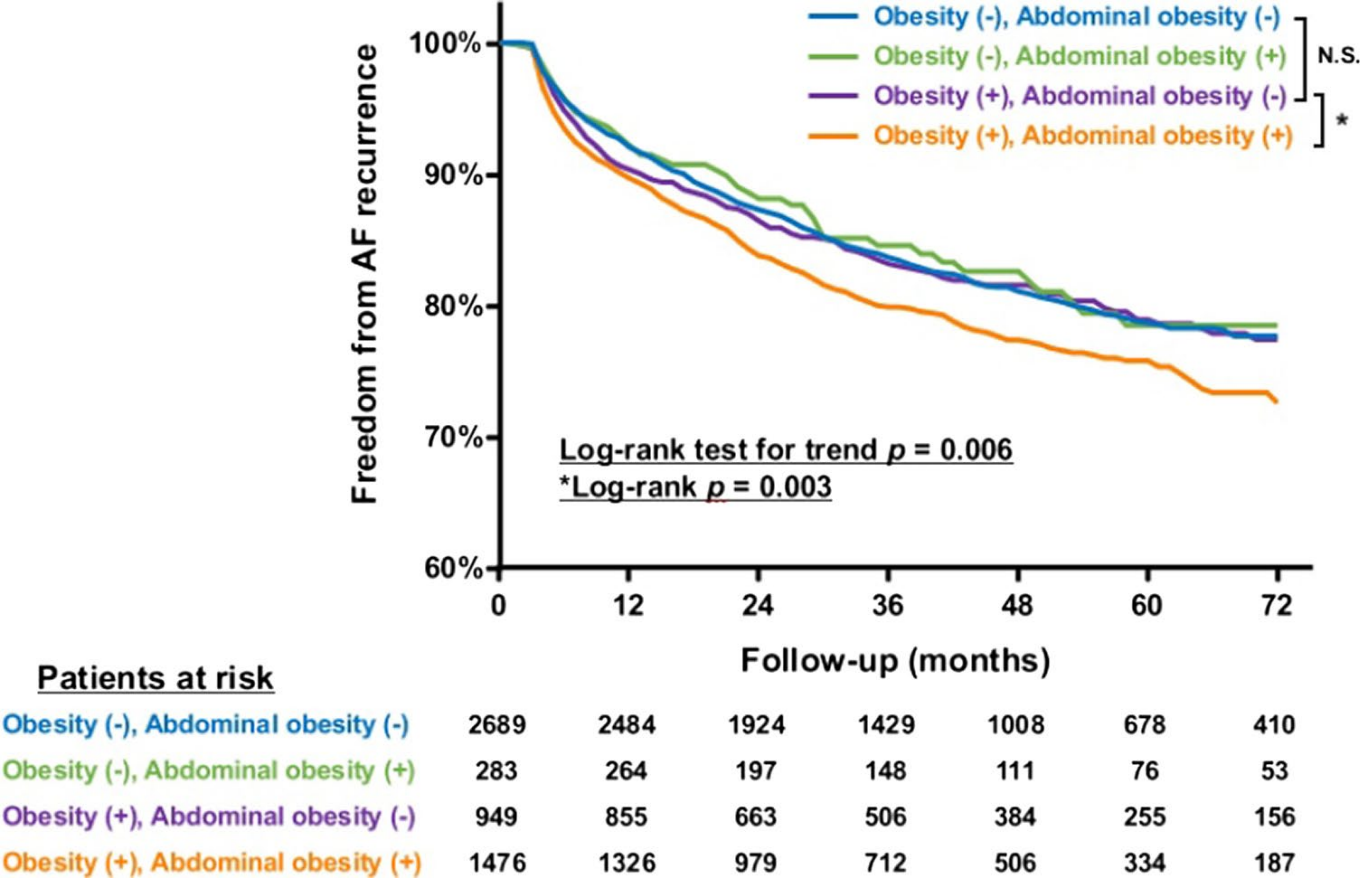
Kaplan‐Meier curve for the effects of obesity (body mass index ≥25 kg/m^2^) and abdominal obesity (waist circumference ≥90 cm for males and ≥85 cm for females) on the long‐term recurrence of atrial fibrillation following index ablation

There was a trend for increased rates of ischemic stroke at 3‐year and 6‐year follow‐ups in patients with abdominal obesity. Furthermore, this group of patients had a greater rate of intracranial haemorrhage. All‐cause death was comparable between both groups. Similar results were obtained for the subset of patients with concomitant heart failure (Table S3), and patients aged less than 65 years old (Table S4). Kaplan‐Meier survival analysis demonstrated significantly higher rates of AF recurrence in patients with obesity based on BMI and waist circumference (log‐rank for trend *P* = .006) (Figure 2). Moreover, among patients with obesity according to BMI, the additional presence of abdominal obesity contributed to an excess of AF recurrence (log‐rank *P* = .003).

**FIGURE 3:**
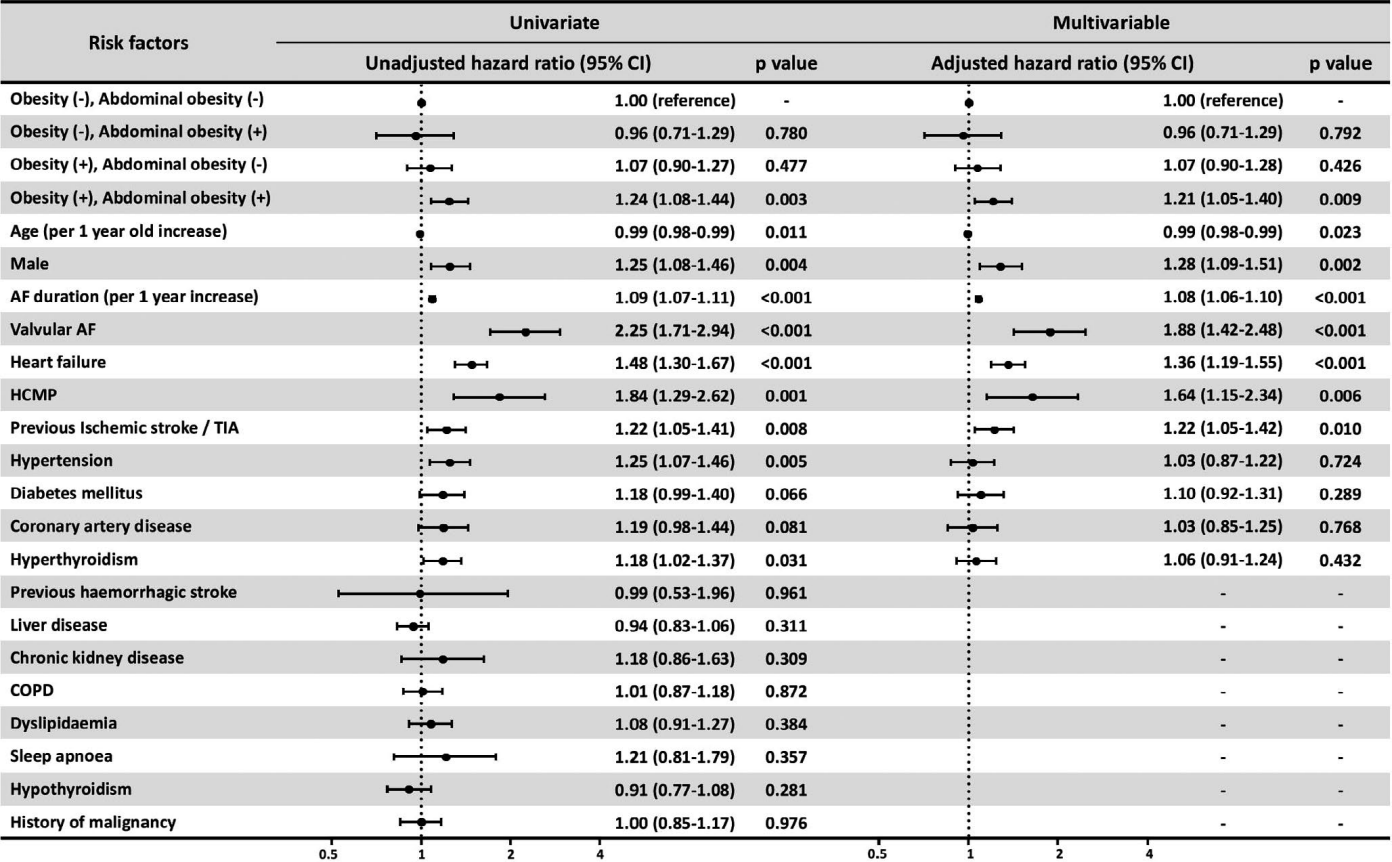
Predictors of long‐term recurrence of atrial fibrillation after index ablation. Multivariable regression model for the outcome of interest was created by including covariates that had univariate significance for the outcome (*P* < .10). Obesity was defined as body mass index ≥25 kg/m^2^ and abdominal obesity defined as waist circumference ≥90 cm for males and ≥85 cm for females. AF, atrial fibrillation; CI, confidence interval; COPD, chronic obstructive pulmonary disease; HCMP, hypertrophic cardiomyopathy; TIA, transient ischemic attack

**FIGURE 4:**
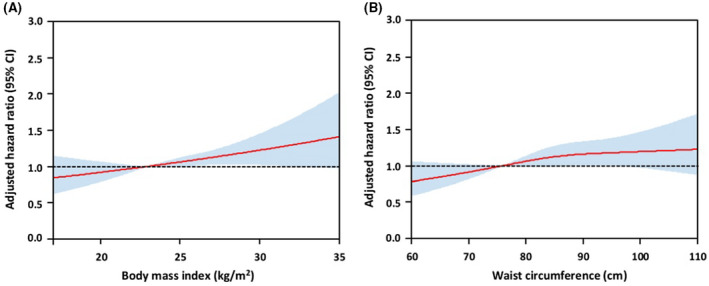
Adjusted hazard ratios for the recurrence of AF after index ablation according to (A) body mass index, and (B) waist circumference. The reference hazard ratios were BMI of 23 kg/m^2^ (A) and waist circumference of 76.2 cm (B). Colour areas are 95% confidence intervals for the spline curves. CI, confidence interval

On multivariable regression analysis, independent predictors for AF recurrence were the presence of obesity by both BMI and waist circumference (hazard ratio [HR] 1.21, 95% confidence interval [CI] 1.05‐1.40), age (HR 0.99 per 1‐year increase, 95% CI 0.98‐0.99), male sex (HR 1.28, 95% CI 1.09‐1.51), AF duration (HR 1.08 per 1‐year increase, 95% CI 1.06‐1.10), valvular AF (HR 1.88, 95% CI 1.42‐2.48), heart failure (HR 1.36, 95% CI 1.19‐1.55), hypertrophic cardiomyopathy (HR 1.64, 95% CI 1.15‐2.34) and previous ischaemic stroke or transient ischaemic attack (HR 1.22, 95% CI 1.05‐1.42) (Figure 3). Plots of adjusted hazard ratios for the recurrence of AF after index ablation according to BMI and waist circumference is shown in Figure 4.

### 3.3 BMI and waist circumference as continuous variables

Multivariable cox regression analyses of BMI and waist circumference as continuous variables demonstrated that both were significantly associated with long‐term recurrence of AF after index ablation (Table 3). Each 1 kg/m^2^ rise in BMI contributed to an adjusted HR of 1.03 (95% CI 1.01‐1.05) and each 1 cm rise in waist circumference contributed to an adjusted HR of 1.01 (95% CI 1.00‐1.02).

**TABLE 3:** Predictors of long‐term recurrence of atrial fibrillation after index ablation including body mass index or waist circumference as a continuous variable

Risk factors	With BMI as a continuous variable				With waist circumference as a continuous variable			
	Univariate		Multivariable		Univariate		Multivariable	
	Unadjusted HR (95% CI)	*P* value	Adjusted HR (95% CI)	*P* value	Unadjusted HR (95% CI)	*P* value	Adjusted HR (95% CI)	*P* value
BMI (per 1 kg/m^2^ increase)	1.04 (1.01‐1.06)	.002	1.03 (1.01‐1.05)	.010	—	—	—	—
Waist circumference (per 1 cm increase)	—	—	—	—	1.01 (1.01‐1.02)	.002	1.01 (1.00‐1.02)	.044
Age (per 1 year increase)	0.99 (0.98‐0.99)	.011	0.99 (0.98‐0.99)	.023	0.99 (0.98‐0.99)	.011	0.98 (0.98‐0.99)	.019
Male	1.25 (1.08‐1.46)	.004	1.28 (1.09‐1.50)	.002	1.25 (1.08‐1.46)	.004	1.22 (1.03‐1.45)	.018
AF duration (per 1 year increase)	1.09 (1.07‐1.11)	<.001	1.08 (1.06‐1.10)	<.001	1.09 (1.07‐1.11)	<.001	1.08 (1.06‐1.10)	<.001
Valvular AF	2.25 (1.71‐2.94)	<.001	1.88 (1.43‐2.49)	<.001	2.25 (1.71‐2.94)	<.001	1.88 (1.42‐2.48)	<.001
Heart failure	1.48 (1.30‐1.67)	<.001	1.36 (1.19‐1.55)	<.001	1.48 (1.30‐1.67)	<.001	1.36 (1.19‐1.55)	<.001
HCMP	1.84 (1.29‐2.62)	.001	1.64 (1.15‐2.34)	.006	1.84 (1.29‐2.62)	.001	1.63 (1.14‐2.32)	.007
Previous ischaemic stroke/TIA	1.22 (1.05‐1.41)	.008	1.22 (1.05‐1.42)	.011	1.22 (1.05‐1.41)	.008	1.22 (1.05‐1.42)	.010
Hypertension	1.25 (1.07‐1.46)	.005	1.03 (0.87‐1.22)	.750	1.25 (1.07‐1.46)	.005	1.04 (0.88‐1.22)	.687
Diabetes mellitus	1.18 (0.99‐1.40)	.066	1.10 (0.92‐1.32)	.286	1.18 (0.99‐1.40)	.066	1.10 (0.92‐1.31)	.290
Coronary artery disease	1.19 (0.98‐1.44)	.081	1.03 (0.85‐1.25)	.766	1.19 (0.98‐1.44)	.081	1.03 (0.85‐1.25)	.761
Hyperthyroidism	1.18 (1.02‐1.37)	.031	1.06 (0.91‐1.24)	.429	1.18 (1.02‐1.37)	.031	1.06 (0.91‐1.24)	.430
Previous haemorrhagic stroke	0.99 (0.53‐1.96)	.961	—	—	0.99 (0.53‐1.96)	.961	—	—
Liver disease	0.94 (0.83‐1.06)	.311	—	—	0.94 (0.83‐1.06)	.311	—	—
Chronic kidney disease	1.18 (0.86‐1.63)	.309	—	—	1.18 (0.86‐1.63)	.309	—	—
COPD	1.01 (0.87‐1.18)	.872	—	—	1.01 (0.87‐1.18)	.872	—	—
Dyslipidaemia	1.08 (0.91‐1.27)	.384	—	—	1.08 (0.91‐1.27)	.384	—	—
Sleep apnoea	1.21 (0.81‐1.79)	.357	—	—	1.21 (0.81‐1.79)	.357	—	—
Hypothyroidism	0.91 (0.77‐1.08)	.281	—	—	0.91 (0.77‐1.08)	.281	—	—
History of malignancy	1.00 (0.85‐1.17)	.976	—	—	1.00 (0.85‐1.17)	.976	—	—

Abbreviations: AF, atrial fibrillation; BMI, body mass index; CI, confidence interval; COPD, chronic obstructive pulmonary disease; HCMP, hypertrophic cardiomyopathy; HR, hazard ratio; TIA, transient ischaemic attack.

### 3.4 Procedural complications of AF ablation

There was a total of 302 (5.6%) reported complications of AF ablation (Table 4). The majority of these were due to pericardial effusion (4.3%). The incidence of stroke or transient ischemic attack, vascular complication requiring intervention and complete atrioventricular nodal block were 0.44%, 0.26% and 0.22%, respectively. Atrio‐oesophageal fistula and phrenic nerve paralysis were extremely rare (<0.1% each).

**TABLE 4:** Impact of abdominal obesity on complications of atrial fibrillation ablation

	Total (n = 5397)	Abdominal obesity (−) (n = 3638)	Abdominal obesity (+) (n = 1759)	*P* value
Total complications, n (%)	302 (5.60%)	216 (5.94%)	86 (4.89%)	.132
Specific complications, n (%)				
Pericardial effusion	232 (4.30%)	172 (4.73%)	60 (3.41%)	.030
Cardiac tamponade	166 (3.08%)	126 (3.46%)	40 (2.27%)	.022
In‐hospital stroke or transient ischemic attack	24 (0.44%)	14 (0.38%)	10 (0.57%)	.464
Vascular complication requiring intervention	14 (0.26%)	8 (0.22%)	6 (0.34%)	.593
Unplanned cardiac or vascular surgery	12 (0.22%)	9 (0.25%)	3 (0.17%)	.800
Complete atrioventricular block	12 (0.22%)	8 (0.22%)	4 (0.23%)	.999
In‐hospital myocardial infarction	7 (0.13%)	3 (0.08%)	4 (0.23%)	.325
Atrio‐oesophageal fistula	5 (0.09%)	5 (0.14%)	0 (0.0%)	.281
Phrenic nerve paralysis	2 (0.04%)	1 (0.03%)	1 (0.06%)	.999
Length of hospital admission (days), mean (SD)	4.93 (2.94)	4.93 (2.83)	4.93 (3.12)	.984

Abbreviation: SD, standard deviation.

Overall complications (abdominal obesity 86 [4.89%] vs 216 [5.94%], *P* = .132) and length of hospital stay (abdominal obesity 4.93 [±3.12] vs 4.93 [±2.83], *P* = .984) were similar in both groups. However, there were significantly less pericardial effusion (3.41% vs 4.73%, *P* = .030) and cardiac tamponade (2.27% vs 3.46%, *P* = .022) among patients with abdominal obesity. The rate of other individual complications was comparable in both groups.

## 4 DISCUSSION

In this study, we present novel results on the effects of abdominal obesity on long‐term outcomes of catheter AF ablation in a large Asian cohort. The main findings were: (a) abdominal obesity was an independent risk factor for long‐term AF recurrence following catheter AF ablation; (b) abdominal obesity was associated with a significantly greater risk of long‐term AF recurrence after index ablation among obese patients by BMI; (c) abdominal obesity was linked to an increased risk of ischaemic stroke and intracranial haemorrhage but not all‐cause death over long‐term follow‐up; and (d) abdominal obesity did not lead to an excess of overall peri‐procedural complications. Furthermore, each 1 cm increase in waist circumference contributed to a 1% increase in the risk of long‐term AF recurrence following ablation.

### 4.1 Impact of abdominal obesity on outcomes of AF ablation

In a prospective study of patients who underwent AF ablation, Shang et al^9^ found that waist circumference was an independent predictor of AF recurrence after adjustment for other risk factors. However, the study comprised only 100 patients with a relatively short follow‐up duration of 13 months. Therefore, we regarded the results as hypothesis‐generating and thus sought to confirm it in a large cohort of patients. Our current findings confirm that abdominal obesity is indeed an independent risk factor for AF recurrence post‐ablation. Nonetheless, this was evident only after 1‐year follow‐up, thus highlighting the importance of long‐term data in this area of research. Potential explanations for this observation include an initial higher threshold for redo ablation and a higher incidence of developing non‐pulmonary vein triggers for AF over time among patients with abdominal obesity.

Despite an increased uptake of anticoagulation in patients with abdominal obesity, there was a trend for higher rates of ischemic stroke in this group. Additionally, these patients were exposed to a greater risk of intracranial haemorrhage. Overall, the greater incidence of AF recurrence in patients with abdominal obesity did not translate to changes in the risk of all‐cause mortality over a 6‐year follow‐up period.

### 4.2 Impact of BMI on outcomes of AF ablation

Unlike abdominal obesity, the effects of BMI as an anthropometric marker of visceral adiposity on outcomes of AF ablation have been evaluated on several occasions. Initial reports were conflicting as some studies demonstrated no association between these factors,^10^ while others found that increased BMI led to a greater risk of AF recurrence post‐ablation.^11^ However, the majority were limited by either a small sample size or a relatively short duration of follow‐up.

A study of 2715 patients undergoing AF ablation found that raised BMI was associated with an increased risk of AF recurrence over five years.^12^ Indeed, two independent meta‐analyses have confirmed that higher BMI is predictive of AF recurrence following ablation therapy.^13^, ^14^ Wong et al^13^ reported that every five‐unit increase in BMI was associated with a 13% excess risk of AF recurrence in the post‐ablation period. However, these results which were based on a majority of studies with a Caucasian population may not be applicable in an Asian cohort. Thus, our study provides useful information in this regard. Here, we found that every unit increase in BMI was associated with a 3% excess risk of long‐term AF recurrence following ablation.

### 4.3 Comparison of abdominal obesity and BMI

Despite evidence supporting the role of BMI as a predictive marker for the outcomes of AF ablation, it has limited accuracy in the diagnosis of obesity and has been shown to “miss” more than half of patients with excess fat.^15^ Furthermore, its implementation may lead to additional misclassification bias in certain populations. For example, Asians tend to have a higher percentage of abdominal adipose tissue for any given BMI compared with Whites.^16^

In terms of clinical events, Hamada et al^17^ found a significantly elevated risk of incident AF with increased waist circumference, independent of BMI and other risk factors. We have also previously demonstrated similar findings that were particularly evident among non‐obese Asian patients.^18^ These results suggest that waist circumference may be superior to BMI for the prediction of incident AF. Moreover, the study by Shang et al^9^ reported that of seven obesity indices, waist circumference was the only independent predictor of AF recurrence following ablation. Our study adds to this as it demonstrates that among patients with obesity by BMI, the additional presence of abdominal obesity was associated with a significantly elevated risk of AF recurrence following ablation. Overall, abdominal obesity as determined by waist circumference is an important marker that may be used to risk stratify patients prior to AF ablation.

### 4.4 Complications of AF ablation

In our study, the total peri‐procedural complication rate was comparable between patients with or without abdominal obesity. Similar findings were reported elsewhere using BMI classification,^19^ although there was the suggestion of increased risk in patients who were morbidly obese (BMI > 40 kg/m^2^).^12^ In terms of individual complications, we demonstrated that patients with abdominal obesity had significantly less pericardial effusion and cardiac tamponade. In contrast, a recent registry study by Friedman et al^20^, found that obesity was linked to an increased risk of cardiac perforation in patients undergoing catheter AF ablation. Reasons for the discrepancy are unclear but warrants further investigation.

### 4.5 Limitations

Our study has several limitations. First, the incidence of AF recurrence was relatively low compared with other studies.^10^ This was likely related to the fact that AF recurrence was determined using rates of cardioversion and repeat ablation. The latter may also provide an explanation for the apparent finding that increasing age was associated with a reduced risk of AF recurrence, as older patients are often deemed less favourable candidates for invasive procedures. Nonetheless, we have proven that this method of assessment had a high positive predictive value for true AF recurrence as determined by electrocardiographic and Holter monitoring (Table S5). Furthermore, as there is a lack of consensus in the definition of AF recurrence and intensity of follow‐up screening employed by various other studies, our approach may be advantageous in that it focuses on clinically important AF recurrence by including only patients with severe symptoms necessitating further intervention. Second, we were unable to account for the effects of AF classification (paroxysmal, persistent and permanent subtypes) and evolution of ablation techniques/tools (such as the use of radiofrequency vs cryoballoon ablation, additional linear ablation, contact force catheter, etc) as these variables were not recorded in the database. Third, the presence of selection bias, misclassification bias and residual confounders cannot be excluded from our study. Fourth, our results may not be applicable to non‐Asian populations.

## 5 CONCLUSIONS

Abdominal obesity as indicated by waist circumference was associated with a greater burden of concomitant diseases and proved to be an independent risk factor for long‐term redo AF intervention following catheter ablation among Korean patients though it had no significant impact on the rate of total peri‐procedural complications. Furthermore, among obese patients by BMI, the additional presence of abdominal obesity contributed to an increased risk of redo AF intervention post‐ablation. Thus, waist circumference may provide a useful, simple marker for clinical risk stratification to guide clinical decision making in patients undergoing AF ablation and serve as a potentially modifiable risk factor to improve long‐term outcomes.

## DISCLOSURES

WYD, PSY, EJ and JHS: None declared. DG: Speaker for Bayer, BMS/Pfizer, Boehringer Ingelheim, Daiichi‐Sankyo, Medtronic, Biosense Webster and Boston Scientific. Proctor for Abbott. Research Grants from Medtronic, Biosense Webster and Boston Scientific. BJ: Speaker for Bayer, BMS/Pfizer, Medtronic, and Daiichi‐Sankyo and received research funds from Medtronic and Abbott. No fees have been received directly/personally. GYHL: Consultant for Bayer/Janssen, BMS/Pfizer, Medtronic, Boehringer Ingelheim, Novartis, Verseon and Daiichi‐Sankyo. Speaker for Bayer, BMS/Pfizer, Medtronic, Boehringer Ingelheim, and Daiichi‐Sankyo. No fees are directly received personally.

## AUTHOR CONTRIBUTIONS

BJ and GYHL contributed to the design of the study. PSY analysed and interpreted the data. WYD interpreted the data and drafted the manuscript. PSY, EJ, JHS, BJ and GYHL revised the manuscript critically for important intellectual content.

## Supporting information

Table S1‐S5

## Data Availability

The data underlying this article will be shared on reasonable request to the corresponding author.
