# Supplementary material for: Impact of abdominal obesity on outcomes of catheter ablation in Korean patients with atrial fibrillation
Source: Int J Clin Pract. 2021 Aug 6;75(10):e14696. doi: 10.1111/ijcp.14696 (PMC11475307; doi:10.1111/ijcp.14696)
Supplement: Supplementary file 1 — Table S1‐S5 [file IJCP-75-e14696-s001.docx]

# Supplementary Materials

**Impact of abdominal obesity on outcomes of catheter ablation in atrial fibrillation**

Short title: Abdominal obesity and outcomes of AF ablation

Wern Yew Ding^1 *^ MRCP

Pil-Sung Yang^2 *^ MD

Eunsun Jang^3^ MS

Dhiraj Gupta^1^ MD

Jung-Hoon Sung^2^ MD, PhD

Boyoung Joung^3 #^ MD, PhD

Gregory Y. H. Lip^1,4 #^ MD

[* joint first authors; ^#^ joint senior authors]

^1^Liverpool Centre for Cardiovascular Science, University of Liverpool and Liverpool Heart & Chest Hospital, Liverpool, United Kingdom; ^2^Department of Cardiology, CHA Bundang Medical Center, CHA University, Seongnam, Republic of Korea; ^3^Department of Cardiology, Severance Cardiovascular Hospital, Yonsei University College of Medicine, Seoul, Republic of Korea; ^4^Aalborg Thrombosis Research Unit, Department of Clinical Medicine, Aalborg University, Aalborg, Denmark

Corresponding author(s):

**Prof Gregory Y H Lip** [gregory.lip@liverpool.ac.uk](mailto:gregory.lip@liverpool.ac.uk)

Full mailing address University of Liverpool

6 West Derby Street

Liverpool, L7 8TX

Telephone number 0151 794 9020

**Prof Boyoung Joung**  [cby6908@yuhs.ac](mailto:cby6908@yuhs.ac)

Full mailing address Yonsei University

50-1 Yonsei-ro, Seodaemun-gu,

Seoul, Republic of Korea 03722

Telephone number 82-2-2228-8460

**Supplementary Table 1.** Definitions and ICD-10 codes used for defining the comorbidities and clinical outcomes.

|  | **Definitions** | **ICD-10 codes or conditions** |
| --- | --- | --- |
| **Comorbidities** |  |  |
| Hypertension | Defined from diagnosis* plus treatment | I10, I11, I12, I13, I15  Treatment: all kinds of blood pressure lowering medications (>1 month). |
| Heart failure | Defined from diagnosis* | I11.0, I50, I97.1 |
| Diabetes mellitus | Defined from diagnosis* plus treatment | E10, E11, E12, E13, E14  Treatment: all kinds of oral antidiabetics and insulin. |
| Previous ischemic stroke | Defined from diagnosis* | I63, I64 |
| Previous TIA | Defined from diagnosis* | G45 |
| Previous myocardial infarction | Defined from diagnosis* | I21, I22, I25.2 |
| Previous intracranial bleeding | Defined from diagnosis* | I60, I61, I62 |
| Peripheral vascular disease | Defined from diagnosis* | I70, I71 |
| Hypertrophic cardiomyopathy | Defined from diagnosis | I42.1, I42.2 |
| Chronic kidney disease | Defined from eGFR or diagnosis* (if laboratory value was not available, diagnosis code was used) | eGFR <60mL/min per 1.73 m2  N18, N19 |
| Chronic obstructive pulmonary disease | Defined from diagnosis* plus treatment | J42, J43(except J43.0), J44  Treatment: SABA, SAMA, LABA, LAMA, ICS, ICS+LABA, or methylxanthine (>1 months). |
| Dyslipidemia | Defined from diagnosis* | E78 |
| Liver disease | Defined from diagnosis of chronic liver disease, cirrhosis, and hepatitis | B18, K70, K71, K72, K73, K74, K76.1 |
| Malignancy | Defined from diagnoses of cancer (non-benign) | C00-C97 |
| Hyperthyroidism | Defined from diagnosis* | E05 |
| Hypothyroidism | Defined from diagnosis* | E03 |
| Anemia | Defined from laboratory data (if laboratory data were available, it was defined) | Hemoglobin concentration <13g/dL in men and <12g/dL in women |
| Dementia | Defined from diagnosis* | F00, F01, F02, F03, G30 |
| Sleep apnea | Defined from diagnosis* | G47.3 |
| **Clinical outcomes** |  |  |
| Cardioversion | Defined from insurance claim procedure code for cardioversion | Procedure code: M5880  (except procedure of M5880 with cardiopulmonary resuscitation) |
| Repeat AF ablation | Defined from insurance claim procedure code for AF ablation with admission diagnosis of AF | Procedure code: M6542, M6547 |
| Ischemic stroke | Defined from admission diagnosis of ischemic stroke with concomitant imaging studies of the brain or related death | I63, I64  Brain-imaging studies include computed tomography or magnetic resonance imaging. |
| Intracranial hemorrhage | Defined from admission diagnosis of ICH with concomitant imaging studies of the brain or related death | I60-I62 |

*To ensure accuracy, comorbidities were established based on one inpatient or two outpatient records of ICD-10 codes in the database.

AF, atrial fibrillation; eGFR, estimated glomerular filtration rate; ICD-10, International Classification of Diseases-10th Revision; ICH, intracranial hemorrhage; ICS, inhaled corticosteroid; LABA, long-acting beta-agonist; LAMA, long-acting muscarinic antagonist; SABA, short-acting beta-agonist; SAMA, short-acting muscarinic antagonist; TIA, transient ischemic attack.

All covariates were validated in cited references.

**Supplementary Table 2.** Definitions of procedural complications of AF ablation.

| **Procedural complications** | **Definition** | **ICD-10 code / procedure code** |
| --- | --- | --- |
| Pericardial effusion | Defined from diagnosis or procedure code for pericardiocentesis within 1 month after AF ablation | I312, I313, I319 / C8060, C8061 |
| Cardiac tamponade | Defined from procedure code for pericardiocentesis within 1 month after AF ablation | C8060, C8061 |
| Vascular complication requiring intervention | Defined from procedure code for vascular intervention or vascular surgery within 3 months after AF ablation | M6644, O2073, O2074, O2035, OA633, OA638, OA639 |
| Complete atrioventricular block | Defined from diagnosis within 1 month after AF ablation | I442 |
| Atrio-esophageal fistula | Defined from diagnosis within 3 months after AF ablation | K223 |
| Phrenic nerve paralysis | Defined from diagnosis within 1 month after AF ablation | J986, G588 |
| Unplanned cardiac or vascular surgery | Defined from procedure code for cardiac or vascular surgery before discharge after AF ablation | O1641, OA641, O1642, OA642, O1647, OA647, O1730, O1740, O1750, O1760, O1770, O1781, O1782, O1783, O1791, O1792, O1793, O1794, O1795, O1796, O1797, O1798, O1660, O1823, O1824, O1825, O1830, O1840, O1970, O1981, O1982, O2006, O2007 |
| In-hospital stroke/TIA | Defined from diagnosis with concomitant imaging studies of the brain before discharge after AF ablation | I63, I64, G45  Brain-imaging studies include computed tomography or magnetic resonance imaging. |
| In-hospital myocardial infarction | Defined from diagnosis with concomitant coronary angiography before discharge after AF ablation | I21, I22 |

AF, atrial fibrillation; ICD-10, International Classification of Diseases-10th Revision; TIA, transient ischemic attack.

Supplementary Table 3. Effects of abdominal obesity on outcomes of atrial fibrillation ablation at 1 and 6 years follow-up according to concomitant HF

|  | **Without heart failure (n=3,492)** | | | | | | | **With heart failure (n=1,905)** | | | | | | |
| --- | --- | --- | --- | --- | --- | --- | --- | --- | --- | --- | --- | --- | --- | --- |
|  | Abdominal Obesity (-) (n=2,440) | | Abdominal Obesity (+) (n=1,052) | | | *p* value | | Abdominal Obesity (-) (n=1,198) | | Abdominal Obesity (+) (n=707) | | | *p* value | |
|  | Number of events | Event rate  (% or per 100 PYs) | Number of events | Event rate  (% or per 100 PYs) |  | | Number of events | | Event rate  (% or per 100 PYs) | Number of events | Event rate  (% or per 100 PYs) |  | |  |
| **Early after AF ablation** |  |  |  |  |  | |  | |  |  |  |  | |  |
| All-cause early mortality^†^ | 5 | 0.2 | 1 | 0.1 | 0.675 | | 3 | | 0.3 | 1 | 0.1 | 0.999 | |  |
| **1-year follow-up** |  |  |  |  |  | |  | |  |  |  |  | |  |
| AF recurrence | 174 | 7.4 | 93 | 9.2 | 0.079 | | 130 | | 11.4 | 79 | 11.9 | 0.789 | |  |
| Major adverse events | 26 | 1.1 | 16 | 1.5 | 0.254 | | 28 | | 2.4 | 23 | 3.3 | 0.231 | |  |
| Ischemic stroke | 18 | 0.7 | 12 | 1.2 | 0.236 | | 25 | | 2.1 | 17 | 2.4 | 0.650 | |  |
| ICH | 2 | 0.1 | 6 | 0.6 | 0.006 | | 1 | | 0.1 | 4 | 0.6 | 0.046 | |  |
| All-cause death | 13 | 0.5 | 3 | 0.3 | 0.321 | | 6 | | 0.5 | 4 | 0.6 | 0.849 | |  |
| **6-year follow-up** |  |  |  |  |  | |  | |  |  |  |  | |  |
| AF recurrence | 388 | 4.5 | 189 | 5.3 | 0.078 | | 251 | | 7.0 | 166 | 8.2 | 0.103 | |  |
| Major adverse events | 88 | 0.9 | 39 | 1.0 | 0.802 | | 57 | | 1.4 | 53 | 2.2 | 0.010 | |  |
| Ischemic stroke | 20 | 0.5 | 23 | 0.6 | 0.744 | | 41 | | 1.0 | 36 | 1.5 | 0.060 | |  |
| ICH | 13 | 0.1 | 10 | 0.2 | 0.147 | | 6 | | 0.1 | 9 | 0.4 | 0.059 | |  |
| All-cause death | 43 | 0.4 | 12 | 0.3 | 0.197 | | 19 | | 0.4 | 13 | 0.5 | 0.640 | |  |

^†^All-cause early mortality was defined as mortality occurring either at index AF ablation admission or within 30 days after ablation. AF, atrial fibrillation; ICH, intracranial hemorrhage; PYs, patient-years.

Supplementary Table 4. Effects of abdominal obesity on outcomes of atrial fibrillation ablation at 1 and 6 years follow-up according to age

|  | **Age < 65 years old (n=4,023)** | | | | | | **Age ≥ 65 years old (n=1,374)** | | | | | |
| --- | --- | --- | --- | --- | --- | --- | --- | --- | --- | --- | --- | --- |
|  | Abdominal Obesity (-) (n=2,744) | | Abdominal Obesity (+) (n=1,279) | | | *p* value | Abdominal Obesity (-) (n=894) | | Abdominal Obesity (+) (n=480) | | *p* value |  |
|  | Number of events | Event rate  (% or per 100 PYs) | | Number of events | Event rate  (% or per 100 PYs) |  | Number of events | Event rate  (% or per 100 PYs) | Number of events | Event rate  (% or per 100 PYs) |  |  |
| **Early after AF ablation** |  |  | |  |  |  |  |  |  |  |  |  |
| All-cause early mortality^†^ | 6 | 0.2 | | 1 | 0.1 | 0.443 | 2 | 0.2 | 1 | 0.2 | 0.999 |  |
| **1-year follow-up** |  |  | |  |  |  |  |  |  |  |  |  |
| AF recurrence | 220 | 8.3 | | 131 | 10.8 | 0.018 | 84 | 9.9 | 41 | 8.9 | 0.610 |  |
| Major adverse events | 36 | 1.3 | | 25 | 2.0 | 0.118 | 18 | 2.0 | 14 | 3.0 | 0.290 |  |
| Ischemic stroke | 29 | 1.1 | | 19 | 1.5 | 0.241 | 14 | 1.6 | 10 | 2.1 | 0.491 |  |
| ICH | 2 | 0.1 | | 5 | 0.4 | 0.024 | 1 | 0.1 | 5 | 1.1 | 0.012 |  |
| All-cause death | 12 | 0.4 | | 3 | 0.2 | 0.326 | 7 | 0.8 | 4 | 0.8 | 0.393 |  |
| **6-year follow-up** |  |  | |  |  |  |  |  |  |  |  |  |
| AF recurrence | 496 | 5.3 | | 273 | 6.7 | 0.003 | 143 | 4.9 | 82 | 5.5 | 0.454 |  |
| Major adverse events | 86 | 0.8 | | 50 | 1.1 | 0.150 | 59 | 1.8 | 42 | 2.5 | 0.120 |  |
| Ischemic stroke | 61 | 0.6 | | 39 | 0.8 | 0.089 | 30 | 0.9 | 20 | 1.2 | 0.404 |  |
| ICH | 13 | 0.1 | | 7 | 0.1 | 0.711 | 6 | 0.2 | 12 | 0.7 | 0.004 |  |
| All-cause death | 31 | 0.3 | | 8 | 0.2 | 0.149 | 31 | 0.9 | 17 | 1.0 | 0.891 |  |

^†^All-cause early mortality was defined as mortality occurring either at index AF ablation admission or within 30 days after ablation. AF, atrial fibrillation; ICH, intracranial hemorrhage; PYs, patient-years.

## Supplementary Table 5. Validation of the definitions for detecting AF recurrence, using the results of ECG/Holter monitoring with a total of 212 patients from a tertiary cardiovascular centre. AF recurrence was defined by cardioversion or redo-ablation beyond a 3 months blanking period.

|  | **Proven recurrence (+)**  **(N=87)** | **Proven recurrence (-)**  **(N=125)** |  |
| --- | --- | --- | --- |
| **AF recurrence (N=43)** | 42 | 1 | **PPV:**  **97.7%** |
| **No AF recurrence (N=169)** | 45 | 124 | **NPV:**  **73.4%** |
|  | **Sensitivity: 48.3%** | **Specificity: 99.2%** |  |
